# Poly(ethylene imine)-chitosan carbon dots: study of its physical–chemical properties and biological in vitro performance

**DOI:** 10.1186/s11671-023-03907-4

**Published:** 2023-10-17

**Authors:** Nicolás Santos, Santiago Valenzuela, Camilo Segura, Igor Osorio-Roman, Macarena S. Arrázola, Concepción Panadero-Medianero, Paula A. Santana, Manuel Ahumada

**Affiliations:** 1 Escuela de Biotecnología, Facultad de Ciencias, Ingeniería y Tecnología, Universidad Mayor, Camino La Pirámide 5750, Huechuraba, Santiago, RM Chile; 2 Instituto de Ciencias Aplicadas, Universidad Autónoma de Chile, El Llano Subercaseaux 2801, San Miguel, Santiago, Chile; 3 Instituto de Ciencias Químicas, Facultad de Ciencias, Universidad Austral de Chile, Isla Teja S/N, Valdivia, Región de los Ríos Chile; 4 Centro de Biología Integrativa, Facultad de Ciencias, Ingeniería y Tecnología, Universidad Mayor, Camino La Pirámide 5750, Huechuraba, Santiago, RM Chile; 5 Centro de Nanotecnología Aplicada, Facultad de Ciencias, Ingeniería y Tecnología, Universidad Mayor, Camino La Pirámide 5750, Huechuraba, Santiago, RM Chile

**Keywords:** Carbon dots, Poly (ethylene imine), Chitosan, Cytotoxicity, Antibacterial activity

## Abstract

Carbon dots (CDs) have been quickly extended for nanomedicine uses because of their multiple applications, such as bioimaging, sensors, and drug delivery. However, the interest in increasing their photoluminescence properties is not always accompanied by cytocompatibility. Thus, a knowledge gap exists regarding their interactions with biological systems linked to the selected formulations and synthesis methods. In this work, we have developed carbon dots (CDs) based on poly (ethylene imine) (PEI) and chitosan (CS) by using microwave irradiation, hydrothermal synthesis, and a combination of both, and further characterized them by physicochemical and biological means. Our results indicate that synthesized CDs have sizes between 1 and 5 nm, a high presence of amine groups on the surface, and increased positive ζ potential values. Further, it is established that the choice and use of different synthesis procedures can contribute to a different answer to the CDs regarding their optical and biological properties. In this regard, PEI-only CDs showed the longest photoluminescent emission lifetime, non-hemolytic activity, and high toxicity against fibroblast. On the other hand, CS-only CDs have higher PL emission, non-cytotoxicity associated with fibroblast, and high hemolytic activity. Interestingly, their combination using the proposed methodologies allow a synergic effect in their CDs properties. Therefore, this work contributes to developing and characterizing CD formulations based on PEI and CS and better understanding the CD’s properties and biological interaction.

**Supplementary Information:**

The online version contains supplementary material available at 10.1186/s11671-023-03907-4.

## Introduction

Nanomaterials applications in biomedicine have promoted outstanding advances within the field, with remarkable results in drug delivery, imaging, sensing, tissue regeneration, and antibacterial, among others [1, 2]. Mainly for this last one, metal-based nanomaterials, such as silver and gold, have been employed, as a gold standard in the field, to treat a broad spectrum of bacterial infections with positive results [3, 4]. However, these nanomaterials are not exempt from some biological preoccupations; for example, it has been described that metal-based nanoparticles could have an adverse biological impact by promoting/exacerbating oxidative stress [5–7]. Also, instabilities derived from their sometimes-poor surface stability can lead to nanoparticle aggregation, promoting clearance problems [8]. Further, they can activate the immune system triggering the inflammation processes and potentially leading to fibrosis or other related diseases [9]. Therefore, new nanomaterials that can improve or replace metal-based nanosystems are highly desired within the biomedical field. Among them, carbon-based nanomaterials, particularly carbon dots (CDs), have caught researchers’ attention [10].

Carbon dots, also known as carbon quantum dots, are quasi-spherical nanoparticles with sizes smaller than 10 nm and possess robust and tunable photoluminescent properties [11]. They have been described as nanosystems with low biological toxicity and a wide range of applications within biomedicine, such as carriers, imaging, and sensors. [12]. There are several described procedures for CD preparation [13]; nonetheless, two bottom-up methodologies have been quickly extended in the literature: microwave irradiation and hydrothermal synthesis. The first one consists of the use of microwave energy which can elevate the solution temperature to 1000 °C within a minute [14] in a homogeneous manner without contact during the heat transfer [15], thus, promoting CDs formation by pyrolysis in a reproducible, quick, and effective way [16]. On the other hand, the hydrothermal procedure involves applying high temperatures and pressures to induce the sample carbonization, requiring economical equipment and low energy in a simple process [17]. Furthermore, both methodologies can be combined in a procedure known as a microwave-assisted hydrothermal method, allowing for a combination of some of the previously mentioned benefits [18]. However, a comparison of the impact on the CD’s physicochemical and biological properties of formulations prepared under individual or combined methodologies must be further explored.

Several reagents, either from the natural or synthetic origin, have been proposed as a carbon source for CDs synthesis [13], where its selection depends on the final application [19] or desired photoluminescent (PL) property [20], to mention a couple. Among them, those rich in nitrogen atoms and positively charged have caught special attention due to their highly tunable PL properties, carrier ability for negatively charged biomolecules, and functionalization, to name a few [21, 22]. In this regard, poly(ethyleneimine) (PEI) has been actively used for CDs synthesis and application in biomedicine; For example, Dou et al. (2015) developed fluorescent CDs based on PEI for successful Gram-positive and Gram-negative bacteria removal and further proved its ability to transport genes [23]. Another highly employed molecule in biomedicine is chitosan (CS), a natural biomacromolecule associated with antibacterial properties, biocompatibility, and desire degradability [24]. Its use for CDs preparation has also been extended in the literature; for example, Chowdhuri et al. (2015) prepared O-carboxymethyl chitosan conjugated with methyl methacrylate CDs via the hydrothermal procedure, finding that the carbon dots have potential cellular imaging and drug delivery applications [25]. However, from the literature, it is noticed that CDs preparation employing CS usually requires the addition of other reagents as passivation agents. In addition to CD’s biocompatibility and carrier abilities, their antibacterial activity properties have quickly attracted researchers’ attention, primarily because of their photosensitizer activity [26], as they promote local reactive oxygen species formation upon excitation and are highlighted as an improvement in selectivity and cytotoxicity over classical dyes [27]. Still, due to its novelty, it has not been extensively explored in the literature for every formulation.

Previously, Sachdev et al. (2014) reported the one-step hydrothermal synthesis of PEI-CS CDs, comparing its properties against a polyethylene glycol-CS CDs formulation prepared under the same conditions. They established that PEI-CS CDs have superior biocompatibility and bioimaging capabilities than the other formulation [28]. Nonetheless, besides their positive results, an in-deep study of the contribution of the based reagents, a comparison between them, and the effect of the synthesis approach on their physical chemistry, biological, and antibacterial properties have been less explored. In this work, we propose synthesizing and characterizing carbon dots using PEI and CS, prepared using microwave radiation, hydrothermal procedure, and a combination of both. Further, their cytocompatibility and antibacterial potential are established in the absence and presence of light. Finally, the influence of the chosen synthesis methodologies over their physical, chemical, and biological properties is discussed.

## Experimental

### Chemicals

Poly (ethylene imine) (PEI, Mw: 20,000 g/mol), Chitosan (CS, M_w_ 190,000–310,000 Da, degree of deacetylation ≥ 75%, cod. 448,877), and glacial acetic acid (cod. 695,092) were purchased from Sigma Aldrich. All the solutions were prepared using MilliQ water obtained from an Adrona CNB1901 Milli-Q Ultrapure water purification system.

### Carbon dots (CDs) synthesis

CDs derived from chitosan (CS), poly (ethylene imine) (PEI), or a combination of CS-PEI were synthesized either by individual microwave irradiation methodology or hydrothermal synthesis or by application of two consecutive steps involving microwave irradiation and hydrothermal synthesis, respectively, considering previously published works [17, 29–32]. All the formulations exposed in this work were prepared using different batches, at least in triplicate. Each procedure is described in detail in the following lines.

#### Microwave synthesis

CS-PEI CDs were prepared by adding 10 mL of a CS dissolution at 2% w/v (dissolved in acetic acid 0.5 M) and 50 mg of PEI dissolved in 3 mL of MilliQ water to a round-bottom flask. Then, the solution was irradiated using a domestic microwave set up at half-power and at intervals of 0.5 min for 8 min. The time intervals were required to cool down the CD’s solution. Both the initial and final dissolution were clear and transparent. The same procedure was followed for individual CS and PEI CDs, reaching the same final volumes. All the CDs formulations were stored at room temperature and covered from light.

#### Hydrothermal synthesis

The hydrothermal synthesis was performed using the same volumes and quantitates exposed in point 2.2.1. The dissolution was introduced in a poly(tetrafluoroethylene) (PTFE) container and, next, put in a hydrothermal reactor and sealed. Then, the system was heated at 90 ºC for 4 h. After the reactor was cooled to room temperature, the samples were recovered in glass vials. All the CDs formulations were stored at room temperature and covered from light.

#### Hydrothermal synthesis microwave assisted

The combination of both procedures was achieved by carrying out the microwave irradiation in the first instance (Sect. "Microwave synthesis") and, once concluded, performing the hydrothermal synthesis as previously described (Sect. "Hydrothermal synthesis"). The hydrothermal reaction produces a transparent solution for PEI CDs (blue emission under UV light) and PEI-CS CDs (green emission under UV light); the formulation CS CDs have a transparent yellow-like color. All the CDs formulations were stored at room temperature and covered from light.

### Characterization

CD’s absorption bands were measured in a UV–Vis spectrophotometer (Jasco V-750). Further, excitation and emission fluorescence spectra were recorded in a Jasco FP-8300 spectrofluorometer. For the excitation spectra, a 20 nm bandwidth slit_ex_ was utilized. In the case of emission spectra, the slit_ex_ and slit_em_ were used at 5 nm. Details on the λ_ex_ and λ_em_ for each CD are mentioned in the respective figure caption.

A Fluorescence spectrophotometer Chronos DFD was used to measure the fluorescence intensity and phase modulation profile for lifetime determination. A 405 nm laser was used to excite all CDs samples. For fluorescence measurement in solution, standard optics was used (90º). On the other hand, fluorescence lifetime determination was performed using a 430 nm filter in the emission band to remove the generated scattering. Further, the Vinci 3.0 Software was utilized for results analysis.

Molecular structure identification was performed by Fourier-Transform infrared (FTIR) spectroscopy utilizing a Spectrum Two FTIR (Perkin Elmer). Before FTIR measurements, samples were lyophilized (BK-FD10, Biobase) to obtain powder-form CDs. Measurements were carried out using an attenuated total reflectance (ATR) sample accessory, with 64 scans per sample.

Transmission electron microscopy (TEM) was utilized to determine the size and morphology of the CDs. Measurements were carried out in a Talos FF200C G2 (Thermo Scientific) at 120 kV. Samples were prepared by depositing 10 µL on an ultrathin carbon film supported on a copper grid (400 mesh, Ted Pella, Inc.). Further, ζ-potential was determined by laser Doppler anemometry using a Zetasizer Nano ZS (Malvern Instruments, UK).

CD’s formulations thermogravimetric analysis was performed in a Simultaneous Thermal Analysis (STA) 8000 equipment (Perkin Elmer). The measurement was performed using a heating ramp of 10 °C/min and recorded from 25 to 1000 °C. Also, 20 mL/min nitrogen flux was employed as a carrier.

### CD’s cytotoxicity evaluation by Live/Dead assay

Mouse embryonic fibroblast NIH/3T3 cells (ATCC, CRL-1658) were seeded on 48-well plates, with a density of 20,000 cells per well, in Dulbecco’s Modified Eagle Medium (DMEM, Gibco 12,800–017), supplemented with 10% fetal bovine serum (SFB; Capricorn Scientific FBS-HI-11A) and 1% penicillin G-streptomycin (Gibco 15,140–122) at 37 °C under a humid atmosphere at 5% CO_2_. Once 80% confluence was reached, cells were treated with 10, 100, and 500 μg/mL of each CD’s formulation for 24 h. After treatment, cells were incubated with 2 µM Calcein-AM (Invitrogen C3100MP) and 1.5 µM propidium iodide (PI, Sigma P4170) in DMEM without phenol red for 30 min at 37 °C. Calcein-positive cells, stained in green, correspond to live cells, while red nuclei are dead cells. Cells were immediately imaged using a 20 × objective with a Dmi8 Leica fluorescence microscope and analyzed by automatic counting with the Analyze Particles tool of the ImageJ software (NIH, USA). Viability was calculated as the percentage of live cells normalized against the total cell number (calcein + PI cells). Plots and statistical analysis were performed with the GraphPad Prism software v8.0, with results obtained from n = 4 independent experiments.

### CD’s hemolytic activity

Hemolytic assays were performed following the previously described methodology by Santander et al. [33]. All procedures involving animals in the study were in accordance with the American Veterinary Medical Association (AVMA) panel report and under the guidelines established by CONICYT 2009 (Chile) and The International Guide for Care and Use of Laboratory Animals. The experimental protocols were approved by the Bioethics and Biosafety Committee of the Universidad Autónoma de Chile (No. BE 05–23), Santiago, Chile. Briefly, red blood cells (RBC) were obtained from a fresh mouse and immediately processed. Then, 1 mL of this RBC was spun at 2000 × g per 10 min at 4 °C. The supernatant was removed, and the RBC pellet was washed thrice with 10 mM PBS. A 5% v/v suspension of erythrocytes (~ 6 × 10^8^cell/mL) and CDs (10, 100, and 500 μg/mL) were prepared with 10 mM PBS. For this, aliquots of 65 μL of blood suspension and 65 μL of CDs solution were incubated at 37 °C for 1 h. The samples were then centrifuged at 3000 × g for 5 min. Aliquots of 80 μL were added to a 96-well cell culture TPP, and the absorbance was determined at 540 nm in a VERSA max microplate reader. 0.5% (v/v) Triton X-100 was used as a positive control (100% hemolysis). PBS was used as a negative control. The assays were performed in triplicate, and Eq. 1 was employed to determine the hemolysis percentage at different CDs concentrations.

1 $$\% {\text{Hemolysis}} = \left[ {\frac{{\left( {A_{{540\;{\text{nm}}}} {\text{of}}\; {\text{CDs}} - A_{{540\;{\text{nm}}}} {\text{of}}\;{\text{PBS}}} \right)}}{{\left( {A_{{540\;{\text{nm}}}} {\text{Triton}} \times 100 - A_{{540\;{\text{nm}}}} {\text{of}}\;{\text{PBS}}} \right)}}} \right] \times 100$$

### Antibacterial activity

Gram-positive *Staphylococcus aureus* (ATCC 25923) and Gram-negative *Escherichia coli* (ML-35) were employed as bacterial models for Minimal Inhibitory Concentration (MIC). Both strains were cultivated in Mueller–Hinton II (M-H II) medium at 37 °C overnight. Then, the culture was diluted 50 times with M-H II medium and incubated at 200 rpm until an exponential phase between 0.3 and 0.6 at OD_600_ was obtained. Afterward, the cells were centrifuged at 6000 rpm for 2 min, and the pellet was washed two times with a buffer solution (1% M-H II in 10 mM phosphate buffer saline (PBS)). Finally, the pellet was resuspended in the same buffer solution and adjust the concentration to introduce a total of 10^6^ CFU/mL (CFU: colony forming unit) for being exposed to serial dilution since 1 mg/mL Carbon Dots samples overnight (12–16 h) in the condition of light (300 Lx) and darkness at 37 °C. The test was realized in triplicate. The minimum inhibitory concentration (MIC) was defined as the lowest concentration of CDs formulations that completely inhibited bacterial growth after overnight incubation at 37 °C [34].

## Results

### Synthesis of CDs based on chitosan and poly(ethylene imine)

**Fig. 1:**
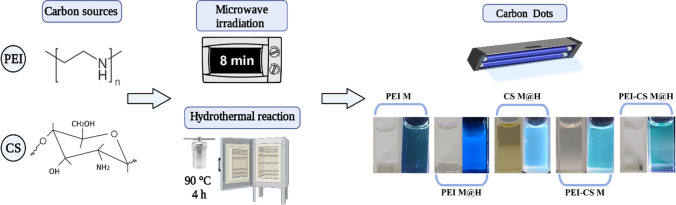
Synthesis of Carbon dots based on chitosan (CS), poly(ethylene imine) (PEI), and PEI-CS. Top, depiction of the followed procedures. Bottom, visual observation of the CDs formulations in the absence (left) and in the presence of UV light (365 nm; right). All the solutions were prepared at the same CD’s final concentration of 1 mg/mL

Carbon dots were synthesized by bottom-up pathways using individual microwave irradiation (M) and hydrothermal (H) methodologies and conjugating both routes as a two-step process (M@H). Figure 1 shows the main synthesis pathways and the obtained CDs solutions in the absence/presence of UV light (365 nm).

Related to the synthesis routes, five of six formulation combinations can produce CDs solutions, where it was not possible to reach chitosan CD solutions by microwave irradiation only means (CS M). Previous studies have reported the synthesis of chitosan CDs using microwave irradiation; however, in all these studies, they used additional reagents to reach a photoluminescence (PL) particle, indicating that chitosan acts as the carbon source while an additional passivation agent is required for CD formation [35–37]. On the other hand, in this work, it was possible to obtain chitosan-only CDs by combining M@H; such results have also been reported and explained in terms of chitosan's ability to self-passivate when is under hydrothermal conditions (high temperature and pressure) [38]. About the PEI, all its formulations were able to produce CDs, as previously reported elsewhere [39–41]. Further, the pictures shown in Fig. 1 is possible to observe differences in colors and PL intensities. While these properties are further explored in the following sections, it is relevant to highlight that in the case of CS M@H’s formulation, it was obtained a brownish color solution. In contrast, the dissolution is translucent for the PEI-CS M@H’s formulation, considering that the starting concentrations are the same in both cases. We associate this effect with PEI, which can stabilize the chitosan molecule, avoiding the carbonization process under working conditions.

### CD’s formulations molecular and physical characterization

**Fig. 2:**
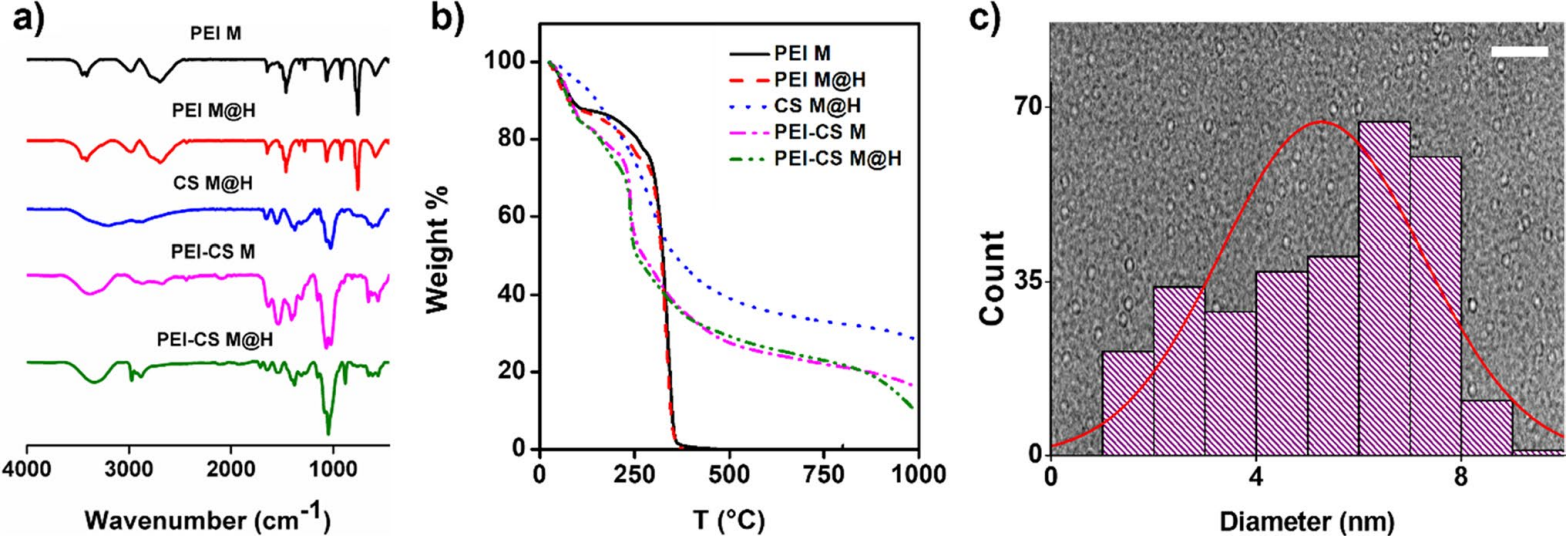
CDs formulation’s composition, thermal, and size analysis. **a** FTIR spectra, each spectrum is accumulative of 64 scans. **b** Thermograms obtained through TGA measurements using a 25–1000 °C sweep and applying a 10 °C ramp. **c** CDs size distribution plot, corresponding to the PEI-CS M@H formulation, was constructed by counting 200 individual particles per sample using TEM images. The red line indicates the main size distribution. The white scale bar corresponds to 50 nm

FTIR was performed to identify the structural composition and presence of functional groups on the surface of the CD’s formulations, which can be observed in Fig. 2A. From the FTIR spectra, it is possible to observe that CD’s formulations retain some of the bands presented in the non-treated reagents (see Fig. S1). Regarding the band’s assignations, characteristics vibrations are observed at 3450–3000, 2980–2700, 1640, 1530, 1461, and 1320 cm^1^, corresponding to N–H/O–H, CH_2_, C=O, N–H, CH_2_, and C-N, respectively [41]. Further, disappearances of the bands at 1570 cm^−1^ for the N–H bending vibration of PEI and that associated with the pyranose ring in chitosan at 880 cm^−1^ have been described as indicative of the CD’s formation [35, 39]. This loss of the pyranose band is attributed to the poor thermal stability of chitosan when subjected to pyrolysis processes [28]. On the other hand, PEI is described as a stable molecule up to 200 °C [42]. TGA analysis was performed to visualize this effect and establish the thermal stability of the proposed formulations (Fig. 2B). Compared to the base reagents (Additional file 1: Fig. S2), all PEI formulations have similar thermal stability up to 320 °C. In this line, CD’s formulations that include CS showed a thermal behavior like the pure chitosan (thermal decays at 80, 310, and over 1000 °C), even in the presence of PEI, which concord with the previously mentioned data. Undoubtedly, the similarity of the thermal behavior of the PEI-CS formulations to the pure chitosan is due to the higher percentage of chitosan within the formulations when compared to the PEI presence. Another relevant observation was that the CS solubility increased after the microwave and/or hydrothermal processes, which can be related to increased hydrophilic functional groups at the surface of the CDs [43].

The CDs sizes distribution was measured based on the obtained TEM images, as depicted in Fig. 2C for the PEI-CS M@H formulation. In the background of Fig. 2C, it is presented a representative TEM image, where a spherical shape for the CDs formulation can be observed, coinciding with previous reports that mention that such morphology is typical among carbon dots formation [44]. Also, the size distribution for all the formulations is shown in Additional file 1: Fig. S3. Nonetheless, the average sizes are presented for each formulation in Table 1. Here, it is possible to conclude that the hydrothermal process increases the size of the obtained CDs, where those of the same composition and subject to both methods are almost twice in size compared to only microwave irradiation synthesis. Previously, Xia et al. (2019) reviewed the carbon dot’s size evaluation process during the synthesis procedure; in that work, high temperatures (as for the microwave technique) can promote a carbon-core formation, while the hydrothermal method at temperatures between 100 and 200 °C contributes to a polymer layer formed on the polymer surface [45]. Thus, this could explain the size increase in our proposed formulations and potentially justify the observed differences in the subsequently evaluated properties. Still, these reported sizes match previously published data for related formulations [28, 35, 37, 46]. Further, there is a size correlation when comparing the individual formulations after the M@H, and the PEI-CS M@H formulations, giving hints that the sizes may be additives. However, such an effect should be further explored in future works.

**Table 1:** CDs formulation’s size, calculated by TEM image, and ζ potential values

Sample	Size (nm)	ζ potential (mV)
PEI M	1.5 ± 0.6	+ 22.30 ± 3.25
PEI M@H	2.8 ± 0.7	+ 9.30 ± 1.41
CS M@H	3.04 ± 1.05	+ 38.90 ± 2.19
PEI-CS M	2.55 ± 0.63	+ 28.63 ± 1.17
PEI-CS M@H	5.27 ± 1.97	+ 38.87 ± 0.84

The measured ζ potentials are also presented in Table 1. ζ potential values correspond to the electrophoretic mobility of colloids, which is generated by the electrical potential of the double layer of the nanoparticle in the presence of an external electric potential [47], where high absolutes values, usually referred to as ≥ ( ±) 25 mV, predict high colloidal stability, as are stabilized by electrical repulsion [48]. Under this consideration, most of the formulations here presented can be considered colloidally stable. Furthermore, as expected, based on the used reagents, all the tested formulations showed a positive ζ value, which can be attributed to the -NH_2_ group [42, 49]. To highlight that the PEI’s ζ potential value decay after the hydrothermal process to almost half, which strongly correlates with the size increase, indicating a decrease in the available amine groups as the surface-to-volume ratio decreases.

### CD’s formulations optical properties characterization

**Fig. 3:**
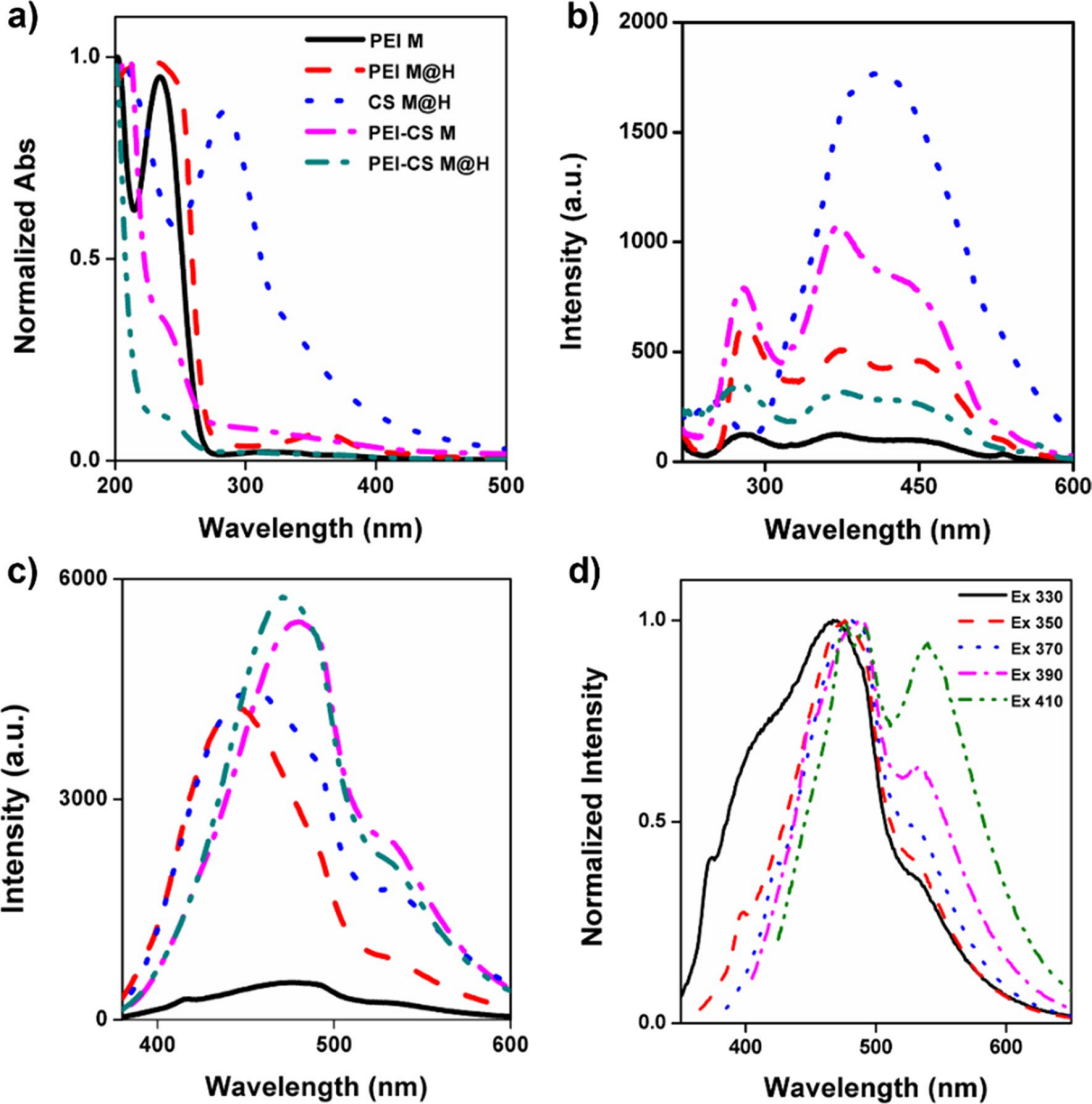
CDs formulations optical properties. **a** normalized UV–Vis’s spectra; **b** excitation spectra; **c** PL emission spectra, CDs were excited at 360 nm. CD’s name formulation description is equivalent from (**a**) to (**c**). **d** PEI-CS M@H’s PL normalized emissions excited at different wavelengths

The most recognizable property of CDs is their optical qualities. We first evaluated their UV–Vis spectra to assess the optical properties of the proposed CDs formulations. Figure 3A shows the UV–Vis spectra, where similar signals were obtained for almost all the formulations. PEI M and PEI M@H showed an intense absorption peak at 230 nm and a small broad peak at 320 nm and 360 nm, respectively. Further, PEI-CS CDs presented similar spectra with shoulder peaks ca. at 240 nm and tails extending up to 400 nm, only differentiating in their absorption values, which can be related to the size increase and functional groups availability at the surface [50]. These observed bands have been previously reported and associated with π − π* and n − π* transitions for conjugated C=C and C=O, respectively [51, 52]. Only the CS M@H formulation showed a different spectrum, with a strong peak centered at 285 nm (associated with π − π* transitions of sp^2^ carbons) [53] and a tail extended over 400 nm, explaining the yellowish color previously described. The excitation spectra, Fig. 3B, had similar behavior to that previously described for the UV–Vis. For all the PEI formulations, excitation peaks at 280, 365, and a shoulder peak at 450 nm are observed. Similarly, for the CS M@H formulation, a strong and broad peak centered at 410 nm was registered. Regarding the PL emission, every formulation shows the same curve shape within the spectra but with differences in their intensities and peak positions, Fig. 3C. PEI M had the lower emission intensity, matching the visual observation in Fig. 1. Further, an increase in emission intensity is observed for the PEI M@H at 445 nm, followed by CS M@H at 453 nm, PEI-CS M at 480 nm, and PEI-CS M@H at 470 nm. This effect in the displacement of the emission band has been previously associated with the sizes (quantum confinement) and defects (or surface energy traps) found on the surface of the CDs [54]. Additionally, Ren et al. [55] had previously shown that the emission spectra obtained for CDs are influenced by their surface chemistry, which in our case, corresponds to a CDs-NH_2,_ which is in line with the previously exposed results. In addition, the PL emission was evaluated at the different excitation wavelengths, showing to be wavelength dependent; see Fig. 3D (corresponding to the PEI-CS M@H formulation as an example). This dependency happens when the excitation energy exceeds the energy gap, and the surface state transitions control the emission [56]. It was also noted that PL emission was almost neglected for almost all the formulations (Fig. S4) after excitation at 410 nm, as reported elsewhere [57]. Finally, from Fig. 3D and those presented in Fig. S4, it can be observed that the emission spectra shape upon red shifting the excitation wavelength change by increasing the intensity at 540 nm from a shoulder peak to an intense peak. Such effect has been previously visualized and described based on the presence of two types of emissive centers; the first is referred to as a protonated state of the molecular groups at the surface, and the second, a deprotonation state, which promotes an energy state red shift by decreasing the energy gap [58], that can be explained in terms of a solvent rearrangement at the excited state depending on the excitation energy. A similar finding was also observed in another study [55].

The CDs formulation’s PL emission lifetimes were also assessed, as shown in Table 2. A tri-exponential decay was observed for all the tested formulations, indicating the presence of different emissive species, which can correspond to fluorophores or energy levels aligned with the previously shown data [59, 60]. Nonetheless, when comparing the average lifetime, it is observed that PEI-only formulations have the longest value and that the CS-only formulation is the shortest. Further, it is interesting to note a lifetime increase as PEI is incorporated with CS after the hydrothermal procedure. This can be explained in terms of the CD’s surface molecular rearrangements. Still, the reported lifetimes here are within the expected durations for carbon dots, as previously reviewed [56].

**Table 2:** CDs formulation’s PL emissions lifetimes*

Sample	τ 1 (ns)	τ 2 (ns)	τ 3 (ns)	Average lifetime (ns)
PEI M	2.84	0.42	10.2	6.34
PEI M@H	2.68	0.39	9.30	6.14
CS M@H	2.42	0.29	9.44	4.04
PEI-CS M	2.86	0.41	9.88	5.07
PEI-CS M@H	3.25	0.52	10.70	6.13

*For complete detail on lifetime measurement, see Table S1

### CDs in vitro cytotoxicity

**Fig. 4:**
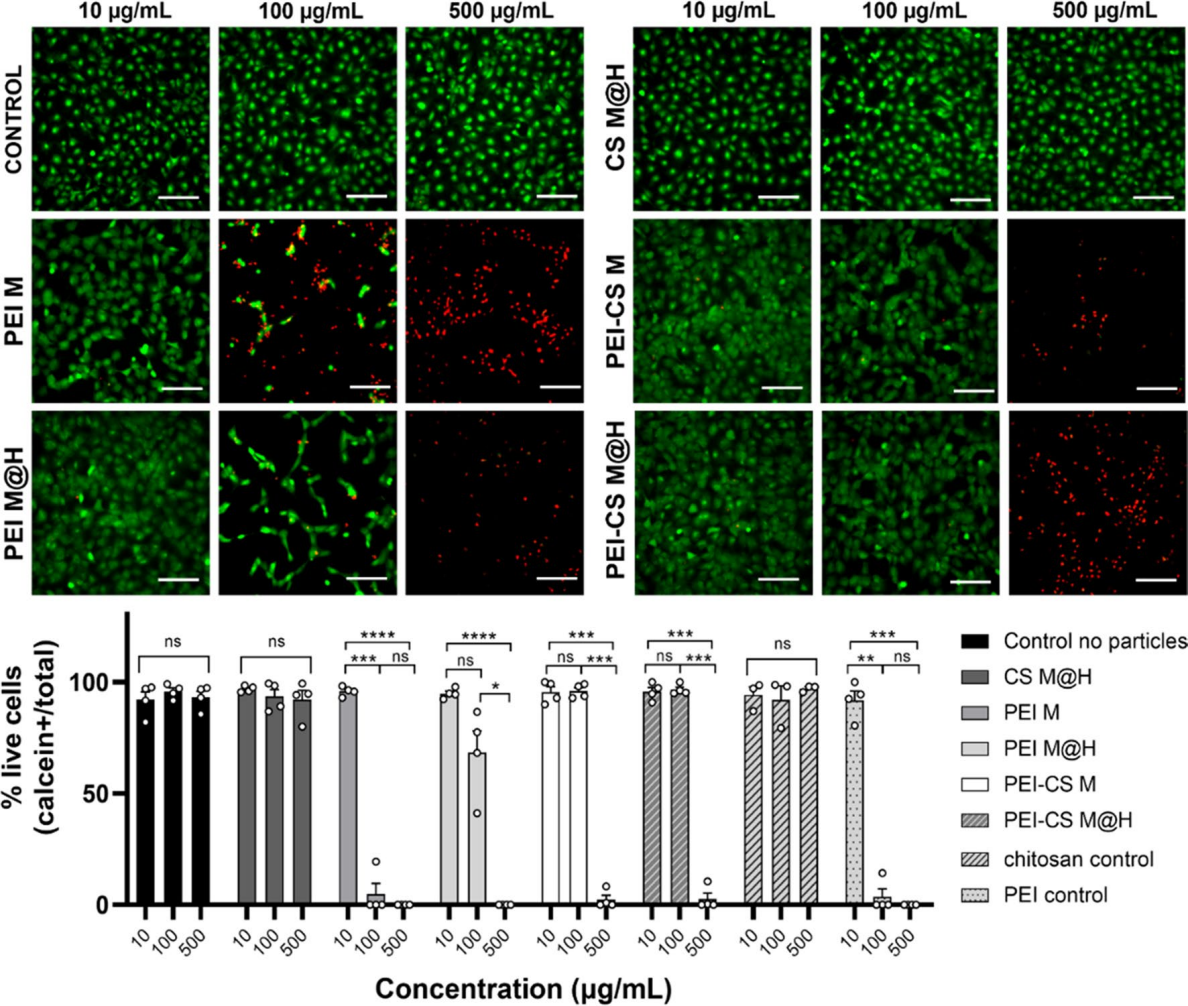
Cytotoxic effects of CD’s formulations in NIH/3T3 fibroblast after 24 h of treatment. Calcein + cells stained in green correspond to live cells. Dead cells correspond to PI + nuclei stained in red)—bars: 150 µm. The percentage of live cells was calculated as the ratio between calcein + cells and the total cell number. Values represent the mean ± SEM. Significance was analyzed by RM-ANOVA and Tukey’s test for multiple comparisons. **: *p* < 0.01, ***: *p* < 0.005, ****: *p* < 0.001

The cytotoxicity of the CDs formulations was tested using NIH/3T3 cell line. Figure 4 shows cell viability results using a live (green)/dead (red) staining procedure after 24 h of treatment. For this work, we tested three CDs concentrations, 10, 100, and 500 μg/mL, chosen based on previous published work using CDs for biological environments [51]. Figure 4 top shows the fluorescence microscopy images obtained for the different tested samples, while the corresponding % of live cells is presented at the bottom. In this figure, it is possible to observe that CDs formulations containing PEI did show loss of viability at concentrations over 10 μg/mL, whereas, for the PEI M, its toxic effect on cell viability was close to 100% at 100 μg/mL (equally as the control PEI polymer); meanwhile, PEI M@H was close to 70% at the same concentration. Nonetheless, both formulations showed 100% cell viability loss at 500 μg/mL. Similar findings were previously described by Havrdova et al. [61] for PEI-based CDs, where the authors found that, for the same cell line, the CDs were highly toxic at concentrations of 100 μg/mL; which was associated with its ability to enter to the nucleus and induce G0/G1/ and G2/M arrest due to their positive charge surface. This effect on the charge can also explain that PEI M@H had a lower toxicity at 100 μg/mL, as it has a lower ζ potential value (Table 1). On the other hand, the CS M@H formulation had a similar result as the controls (ca. 100% viability). Interestingly, when both reagents were combined, viability held close to 100% at 100 μg/mL (in contrast to PEI-only formulations), even though the ζ potential value was higher for PEI-CS formulations; results that align with previous data that highlight the biocompatibility properties of chitosan incorporation into CDs formulations [62, 63]. Yet, both PEI-CS formulations were highly toxic at 500 μg/mL.

### CDs formulations hemolytic activity

**Fig. 5:**
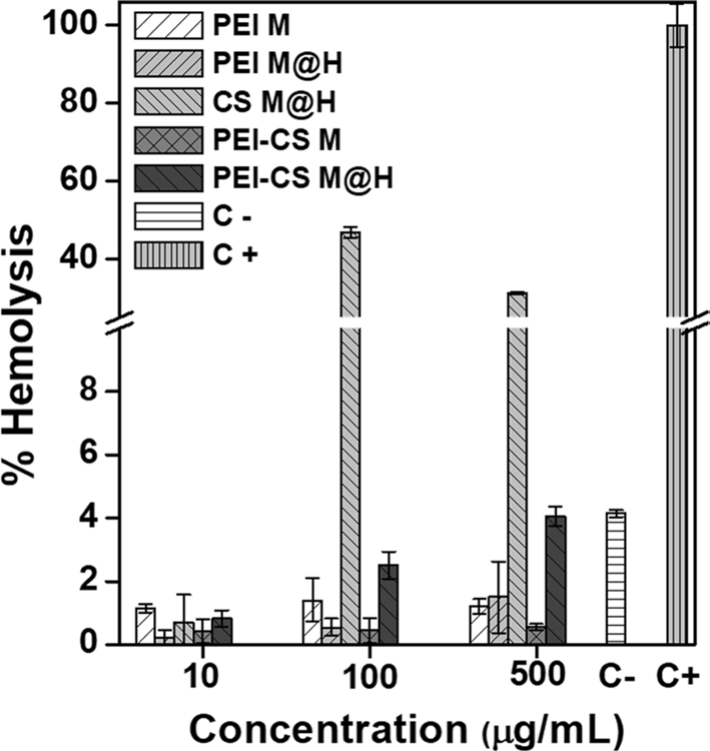
Hemolytic activity of different CDs formulations. Hemolysis (%) of mouse red blood cells (RBCs) treated Carbon Dots derived from chitosan (CS), poly(ethylene imine) (PEI), or a combination of CS-PEI at 10, 100, and 500 μg/ml. RBCs with CDs solutions were incubated at 37 °C for 1 h

To further evaluate the CDs formulation’s biocompatibility, their hemolytic activity was measured to establish any potential cytotoxic activity of the formulations related to cell membrane damage (Fig. 5). In this assay, it was observed that the erythrocyte membrane in the positive control group treated with 0.5% Triton X-100 was disrupted, and hemoglobin flowed out of the erythrocytes, turning red the solution (100% hemolysis). In contrast, hemolysis did not occur in the PBS 1X pH 7.4 solution used as a negative control. By treating the erythrocytes with different CDs formulations at concentrations of 10, 100, and 500 µg/mL, in most samples, the erythrocytes sunk to the bottom, while the upper liquid was colorless and transparent (data not shown). In general, the hemolysis rates of the different CDs formulations were lower than 5% except for CS M@H at 100 and 500 µg/mL, which showed elevated hemolysis rates of 46.8 and 31.3%, respectively. Regarding this hemolytic activity demonstrated by the chitosan formulation, it was not possible to find any previous report of CS-only carbon dots, as all the reported data combine this biopolymer with other reagents. Nonetheless, prior work by de Lima et al. [64] has stated that CS nanoparticles (NPs) can promote higher hemolytic activity due to the acetylation degree and the solution pH. On the contrary, Jesus et al. [65] recently published that no such hemolytic activity is related to CS NPs; however, they noted a decrease in cell viability of macrophages RAW 264.7 cell line at high CS NPs concentration. Still, another work associated the hemolytic activity of CS to the amino groups on the surface of the biopolymer, which, by electrostatic interactions with the red blood cells (RBCs) surface (negatively charged), can induce modification on the membrane curvature and lastly, to lysis, where also the possibility of polyelectrolyte complexes formation was considered [66]. While this last explanation can be durable, it does not necessarily explain why most of the other CDs formulations proposed here did not show hemolysis on the RBCs. Yet, for the PEI-CS M@H formulation, a minimal hemolytic activity was also displayed in a concentration dependency manner. Therefore, while additional work is necessary to uncover the complete hemolytic mechanism, a plausible explanation is that a limit ζ potential value exists to employ nanosystems with RBCs safely. To continue with the hemolytic analysis, as described by Totea et al. (2014), materials that show over 5% hemolysis are considered hemolytic, between 5 and 2% are considered slightly hemolytic, and below 2% are non-hemolytic materials [67]. Therefore, PEI M, PEI M@H, PEI-CS M, and PEI-CS M@H formulations are considered hemocompatible or non-cytotoxic at the concentrations evaluated. The results obtained in this study correlate with those reported by other authors, where CDs obtained through a hydrothermal procedure [68] or Chinese medicinal materials (plants and animals) used as carbon sources [69] were evaluated at concentrations similar to those used in this study (10, 100 and 500 μg/mL), observing a hemolytic activity lower than 5% indicating that they are biocompatible and has potential applications in a biological system.

### CDs antibacterial activity

The antibacterial activity of each proposed formulation was tested at concentrations between 8 and 1000 μg/mL against *E. coli* (Gram-negative bacteria) and *S. aureus* (Gram-positive bacteria) and expressed in terms of MIC (Table 3). For PEI M and PEI M@H, the MICs were between 8 and 16 μg/mL after the susceptibility tests, representing an excellent antimicrobial activity, mainly against *S. aureus* than *E. coli* in the presence and/ the absence of light. The CS M@H formulation showed the same MIC (50 μg/mL) against the two bacteria strains under both light conditions. Similar results were observed for PEI M and PEI M@H against *S. aureus,* with MIC values of 7.81 and 15.6 μg/mL, respectively. Further, it was found that the MIC of PEI-CS M@H formulation, in the presence of light, was lower against *E. coli* (125 μg/mL) than for *S. aureus* (31.2 μg/mL). Similar behavior was observed for PEI-CS M formulation against *S. aureus* (MIC of 31.2 μg/mL) and PEI M (15.6 μg/mL), and PEI M@H (62.5 μg/mL) formulations against *E. coli*. For PEI-CS M@H formulation, no effects on bacteria growth were observed without light, so the MIC value could not be determined (Table 3). Previous studies have reported the CDs' ability to inhibit bacterial growth or kill bacteria through complex mechanisms [70–72], using as a model Gram-positive or Gram-negative bacterial strain, including *E. coli* or *S. aureus* [23, 73]. When comparing our results with those reported studies, the CDs formulations used in this work can efficiently inhibit the growth of bacteria even at concentrations lower than those reported for *E. coli* (MIC 16 and 64 μg/mL) and *S. aureus* (MIC 16 and 32 μg/mL) [23].

**Table 3:** Minimum inhibitory concentration (MIC) values in µg/mL of different CDs formulations in the presence and the absence of light (room light)

Sample	MIC (µg/mL)			
	*E. coli*		*S. aureus*	
	Daylight	Darkness	Daylight	Darkness
PEI M	15.6	500	7.81	7.81
PEI M@H	62.5	250	15.6	15.6
CS M@H	50	50	50	50
PEI-CS M	50	50	31.2	62.5
PEI-CS M@H	125	> 1,000*	31.2	> 1,000*

*For the PEI-CS M@H formulation, the MIC for both tested bacteria is greater (>) than 1,000

Regarding the observed differences between exposition or absence of light, the antibacterial mechanism in darkness is associated with CDs penetration (without cell disruption) and coupling to DNA or RNA, destroying their secondary structure [74, 75]. Meanwhile, in the presence of light, their activity is associated with their ability to transfer excited electrons to other species within the environment (such as O_2_) to form reactive oxygen species (ROS) that, ultimately, produce an oxidative stress cascade within the bacteria that cause its elimination [26]. In this case, the presented results indicated that daylight exposition has a more significant antibacterial effect than in the absence of light. While many factors could contribute to their differences, a plausible explanation for the observed tendency could be related to the measured sizes [76]. Related to the CD’s improved activity against *S. aureus* than *E. coli*, a previous study showed that Gram-positive bacteria have higher negative charge density favoring the electrostatic interaction with the positive charge CDs. In addition, they also highlighted that for Gram-negative bacteria, cell wall penetration is harder for CDs [77]. Similar findings have also been described for glycerol-Si-QAC CDs [78].

## Conclusions

In this work, the synthesis, by microwave irradiation and hydrothermal microwave-assisted procedure, and characterization of CDs based on PEI, CS, and PEI-CS were presented and physicochemical and biologically compared. Related to their physical–chemical properties, all the formulations showed a high presence of amine groups on their surface, which was also corroborated by the highly positive values of ζ potential; nonetheless, observed differences were attributed to their size variations that can be explained in terms of polymers formations on the surface that will vary upon the used reagent. Further, CDs formulations containing CS did show higher PL emission than PEI-only CDs; however, these last ones have longer fluorescent lifetimes, where its mixture with CS also supports the increase in its lifetime. Regarding their biological properties, mixed results were found. On the one hand, PEI-only CDs were toxic against NIH/3T3 fibroblasts, which can be reduced by incorporating CS within the mixture. On the other, CS-only CDs showed high hemolytic activity that can be reduced by PEI addition. While the toxicity mechanisms for PEI and CS are different, they can be finely tuned to match specific biological requirements for biomedical applications. Furthermore, most of the CD’s formulations showed antibacterial activity against *E. coli* and *S. aureus*, with a higher registered activity in the presence of daylight, which is attributed to their photosensitizing capacity. In addition, some of the lower MIC values reported for CDs formulations are presented. While there is room to explore new formulations and mechanisms, particularly those referred to biological interaction, this work presented an in-deep study on the synthesis and characterization of PEI-CS CDs, and it is a step further in the understanding of the biological interaction of this class of CDs and how to finetune a formulation for biomedical applications.

### Supplementary Information

Additional file1 (DOCX 3269 KB)

## Data Availability

The datasets used and/or analyzed during the current study are available from the corresponding author on reasonable request.
