# Supplementary material for: Poly(ethylene imine)-chitosan carbon dots: study of its physical–chemical properties and biological in vitro performance
Source: Discov Nano. 2023 Oct 17;18(1):129. doi: 10.1186/s11671-023-03907-4 (PMC10581970; doi:10.1186/s11671-023-03907-4)
Supplement: Supplementary file 1 — Additional file1 (DOCX 3269 KB) [file 11671_2023_3907_MOESM1_ESM.docx]

**Supporting Information**

**Poly(ethylene imine)-Chitosan Carbon Dots: Study of its physical-chemical properties and biological *in vitro* performance**

Nicolás Santos,^a^ Santiago Valenzuela,^b^ Camilo Segura^c^ , Igor Osorio-Roman^c^ Macarena S. Arrázola,^d^ Concepción Panadero-Medianero,^d^ Paula A. Santana,^b*^ Manuel Ahumada^a,e*^

^a^Escuela de Biotecnología, Facultad de Ciencias, ingeniería y Tecnología, Universidad Mayor, Camino La Pirámide 5750, Huechuraba, Santiago, RM, Chile.

^b^Instituto de Ciencias Químicas Aplicadas, Universidad Autónoma de Chile, el Llano Subercaseaux 2801, San Miguel, Santiago, Chile.

^c^ Instituto de Ciencias Químicas, Facultad de Ciencias, Universidad Austral de Chile, Isla Teja s/n, Valdivia, Región de los Ríos, Chile.

^d^Centro de Biología Integrativa, Facultad de Ciencias, Ingeniería y Tecnología, Universidad Mayor, Camino La Pirámide 5750, Huechuraba, Santiago, RM, Chile.

^e^Centro de Nanotecnología Aplicada, Facultad de Ciencias, Ingeniería y Tecnología, Universidad Mayor, Camino La Pirámide 5750, Huechuraba, Santiago, RM, Chile.

Corresponding authors’ email: [paula.santana@uautonoma.cl](mailto:paula.santana@uautonoma.cl); [Manuel.ahumada@umayor.cl](mailto:Manuel.ahumada@umayor.cl)

Supporting information index

| **Page** |  | **Description** |
| --- | --- | --- |
| S1 | …………………………………………………………….. | This page |
| S2 | …………………………………………………………….. | Figure S1 |
| S3 | …………………………………………………………….. | Figure S2 |
| S4 | …………………………………………………………….. | Figure S3 |
| S5 | …………………………………………………………….. | Figure S4 |
| S6 | …………………………………………………………….. | Table S1 |





**Figure S1.** FTIR spectra of starting reagents; chitosan (CS; black line) and poly(ethylene imine) (PEI; red line). Each spectrum is accumulated of 64 scans.


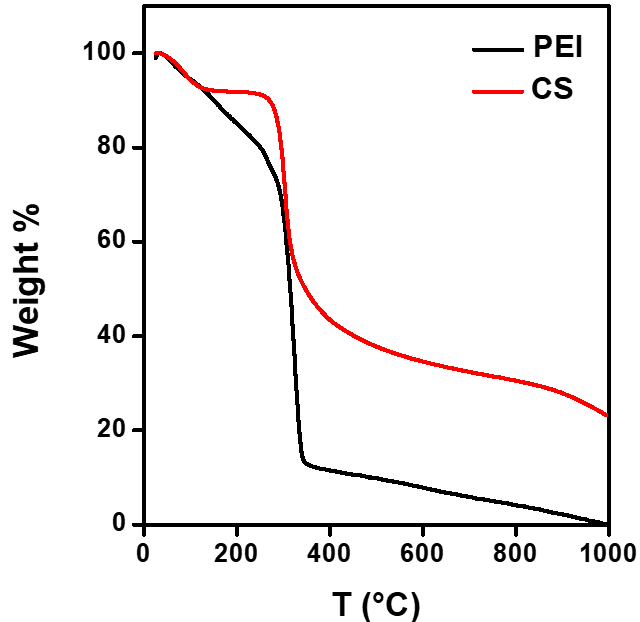


**Figure S2.** TGA thermograms of starting reagents: chitosan (CS; black line) and poly(ethylene imine) (PEI; red line).


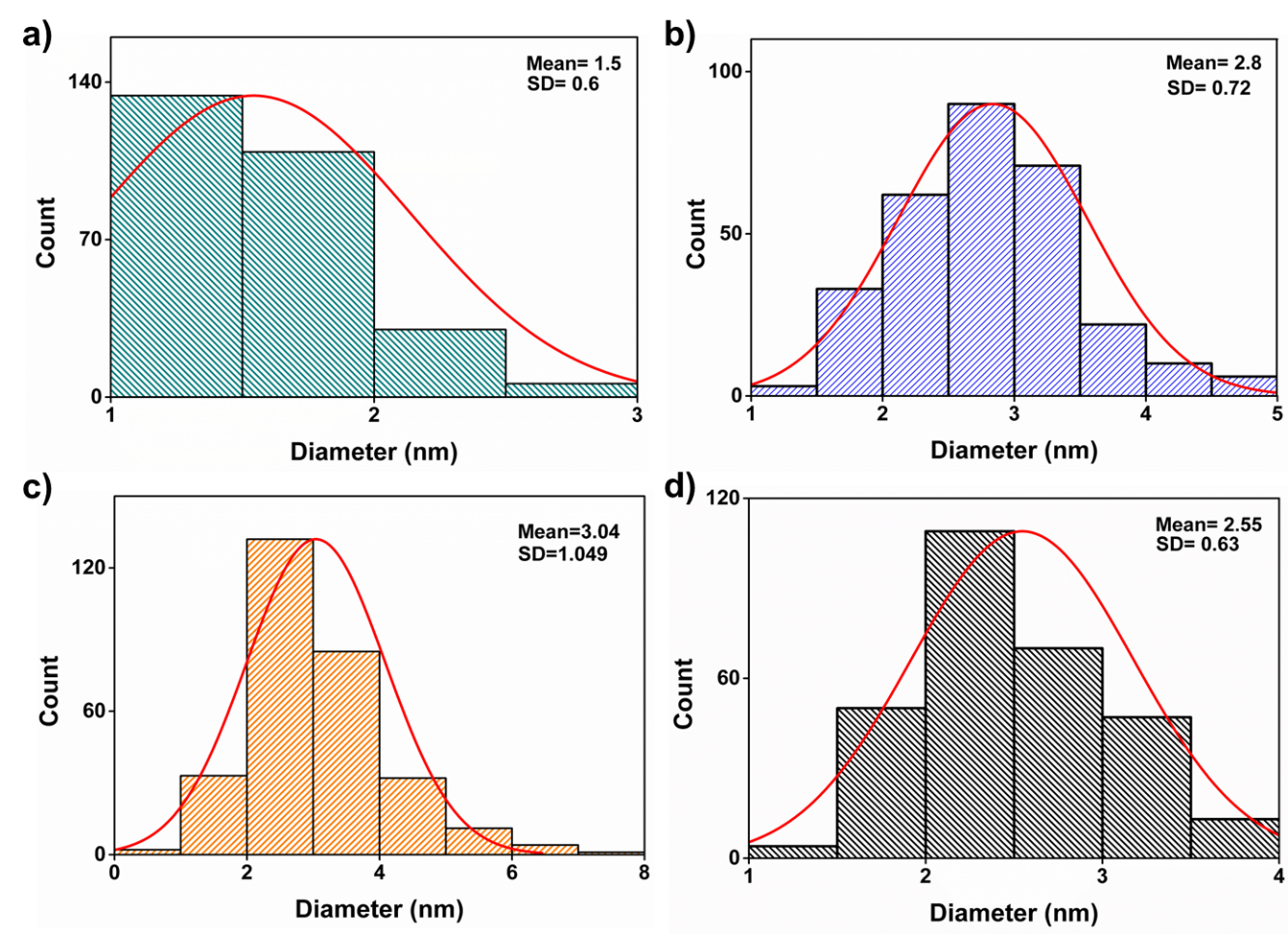


**Figure S3.** CDs size distribution plots. a) PEI M; b) PEI M@H; c) CS M@H; d) PEI-CS M formulations. Plots were constructed by counting 200 individual particles per sample using TEM images. The red line indicates the main size distribution.


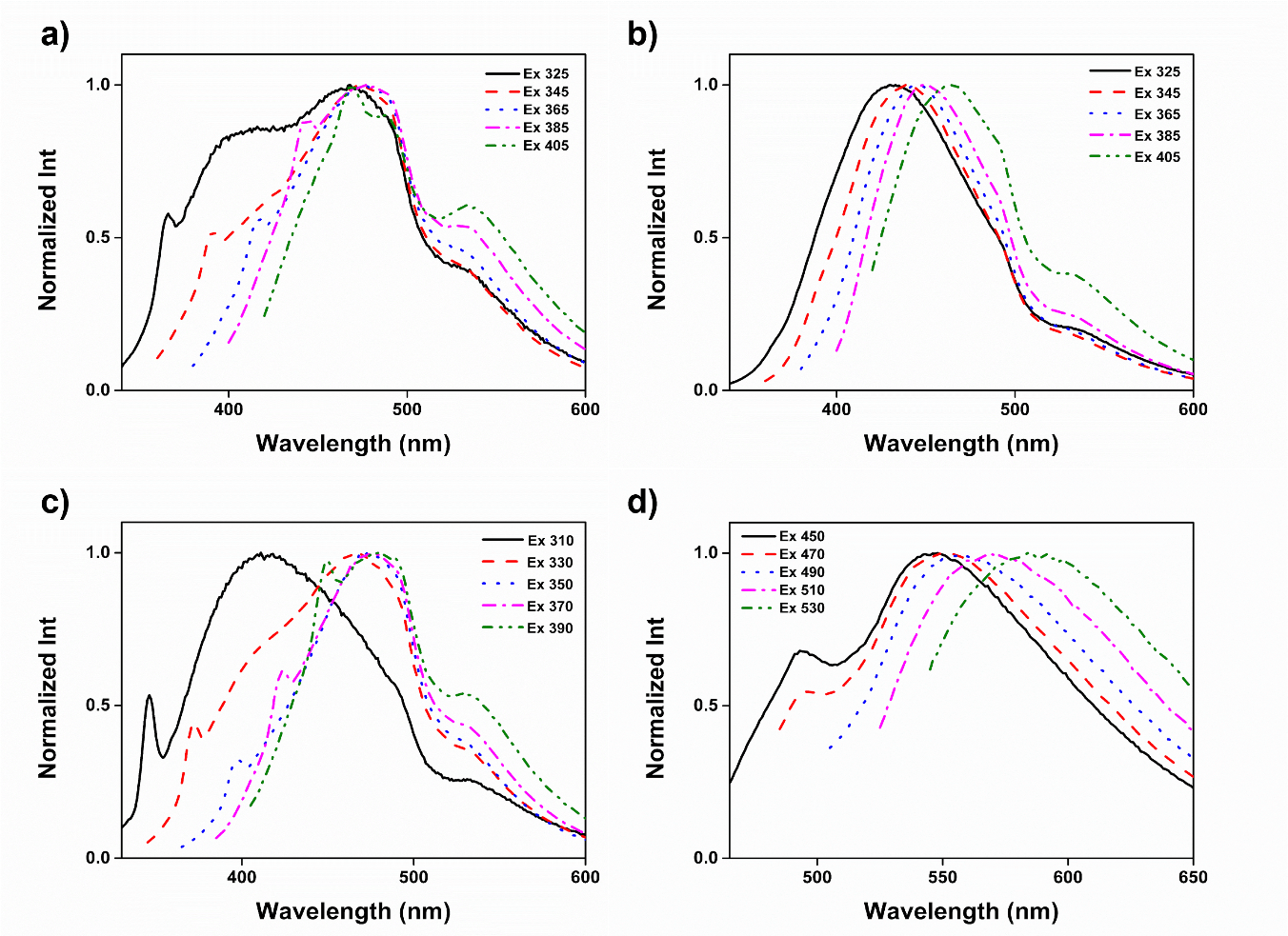


**Figure S4.** PL-normalized emissions were excited at different wavelengths. a) PEI M; b) PEI M@H; c) CS M@H; d) PEI-CS M.

**Table S1.** CDs formulation´s PL emissions lifetimes.

| **Sample** | **Lifetime** | **ns** | **sd** | **%** | **Average lifetime (ns)** | **χ2** |
| --- | --- | --- | --- | --- | --- | --- |
| PEI M | τ 1 | 2.84 | ± 0.10 | 34.6 | 6.34 | 1.03 |
|  | τ 2 | 0.42 | ± 0.02 | 13.5 |  |  |
|  | τ 3 | 10.2 | ± 0.30 | 51.9 |  |  |
| PEI M@H | τ 1 | 2.68 | ± 0.10 | 34.7 | 6.14 | 0.982 |
|  | τ 2 | 0.39 | ± 0.04 | 9.7 |  |  |
|  | τ 3 | 9.30 | ± 0.20 | 55.6 |  |  |
| CS M@H | τ 1 | 2.42 | ± 0.09 | 42.9 | 4.04 | 2.8 |
|  | τ 2 | 0.29 | ± 0.01 | 26.1 |  |  |
|  | τ 3 | 9.44 | ± 0.40 | 31 |  |  |
| PEI-CS M | τ 1 | 2.86 | ± 0.10 | 42.1 | 5.07 | 2.05 |
|  | τ 2 | 0.41 | ± 0.02 | 19.6 |  |  |
|  | τ 3 | 9.88 | ± 0.40 | 38.3 |  |  |
| PEI-CS M@H | τ 1 | 3.25 | ± 0.10 | 43.1 | 6.13 | 2.1 |
|  | τ 2 | 0.52 | ± 0.02 | 13.4 |  |  |
|  | τ 3 | 10.70 | ± 0.40 | 43.5 |  |  |
